# Osteotropism of neuroendocrine tumors: role of the CXCL12/CXCR4 pathway in promoting EMT *in vitro*

**DOI:** 10.18632/oncotarget.15122

**Published:** 2017-02-06

**Authors:** Mauro Cives, Davide Quaresmini, Francesca Maria Rizzo, Claudia Felici, Stella D'Oronzo, Valeria Simone, Franco Silvestris

**Affiliations:** ^1^ Department of Biomedical Sciences and Human Oncology, University of Bari “A. Moro”, Bari, Italy

**Keywords:** carcinoid, bone metastasis, SDF-1, epithelial-mesenchymal transition, GPCR nuclear translocation

## Abstract

Neuroendocrine tumors (NETs) metastasize to the skeleton in approximately 20% of patients. We have previously shown that the epithelial-mesenchymal transition (EMT) regulates the NET osteotropism and that CXCR4 overexpression predicts bone spreading. Here, we unravel the molecular mechanisms linking the activation of the CXCL12/CXCR4 axis to the bone colonization of NETs using cell lines representative of pancreatic (BON1, CM, QGP1), intestinal (CNDT 2.5), and bronchial origin (H727). By combining flow cytometry and ELISA, BON1, CM and QGP1 cells were defined as CXCR4^high^/CXCL12^low^, while H727 and CNDT 2.5 were CXCR4^low^/CXCL12^high^. CXCL12 was inert on cell proliferation, but significantly increased the *in vitro* osteotropism of CXCR4^high^/CXCL12^low^ cells, as assessed by transwell assays with or without Matrigel membranes. In these cells, CXCL12 induced *in vitro* a marked EMT-like transcriptional shift with acquirement of a mesenchymal shape. The nuclei of CXCR4^high^/CXCL12^low^ NET cells were typically enriched in non-phosphorylated CXCR4, particularly upon agonist stimulation. Silencing of CXCR4 via siRNA prevented the CXCL12-induced EMT in CXCR4^high^/CXCL12^low^ NET cell lines resulting in the abrogation of both migration and transcriptional mesenchymal patterns. Our data suggest that CXCL12 conveys EMT-promoting signals in NET cells through CXCR4, which in turn regulates transcriptional, morphologic and functional modifications resulting in enhanced *in vitro* osteotropism of NET cells. Unique functions of CXCR4 may be segregated in relation to its subcellular localization and may acquire potential relevance in future *in vivo* studies.

## INTRODUCTION

Neuroendocrine tumors (NETs) are heterogeneous malignancies arising from the diffuse neuroendocrine system. They are characterized by a relatively indolent rate of growth and share the ability to produce a variety of peptide hormones and vasoactive amines. Although NETs may develop in almost all organs, they are prevalent within the lung, the pancreas and the gastrointestinal tract, and their incidence has markedly increased over the last three decades [1, 2]. Up to 20% of patients with NETs are diagnosed with bone metastases, whose incidence and negative influence on both tumor morbidity and patient prognosis have been recently reported by our group as well as by others [3–5]. In relation to the natural history of NETs, the detection of bone involvement within 3 months from the primary NET diagnosis is apparently associated with a very dismal survival of only 12 months, and such a poor clinical outcome may be the epiphenomenon of an underlying peculiarly aggressive disease [3].

We have previously demonstrated that the epithelial-mesenchymal transition (EMT), a transdifferentiation program that promotes changes of the cell state conferring mesenchymal plasticity to epithelial cells [6], is overactive in bone-colonizing NETs [3]. In this context, overexpression of C-X-C chemokine receptor type 4 (CXCR4) in primary tumors appeared highly predictive of skeletal metastases. Both CXCR4 and its ligand C-X-C motif chemokine 12 (CXCL12) act as positive regulators of the invasion-metastasis cascade, and their pro-tumor effects have been attributed to i) autocrine mechanisms promoting both proliferation and angiogenesis; and ii) paracrine mechanisms leading to the recruitment of CXCR4^+^ cancer/immune/stromal cells to CXCL12-overexpressing organs (i.e., bone, liver and lung), resulting in the formation of the so-called pre-metastatic niche [7, 8].

In NETs, the functional role of the CXCL12/CXCR4 synapse has been scantily investigated. As compared with normal tissue, both CXCL12 and CXCR4 are overexpressed in NETs, where they signal through the mTOR pathway reinforcing the uncontrolled cell growth [9]. Amongst lung, pancreatic and ileal NETs, increased levels of CXCR4 seem to correlate with higher tumor malignancy and are associated with poor patient outcomes [10–12]. Global gene expression profiling of GOT1 ileal carcinoid cells revealed a marked upregulation of CXCR4 in response to hypoxia, and the agonist stimulation of CXCR4 was able to activate the mitogen-activated protein kinase (MAPK) p42/44 pathway, resulting in increased tumor cell migration [13].

How NET cells acquire the ability to metastasize and how organ-specific metastatic traits arise in primary tumors are still unanswered questions. This study was aimed at investigating the functional role of the CXCL12/CXCR4 axis in modulating the bone tropism of NETs in *in vitro* experimental models, and depicts potential future applications for NET treatment by inhibiting the CXCR4-driven EMT as a crucial step of the metastatic process.

## RESULTS

### CXCR4 and CXCL12 are differentially expressed in NET cell lines

By flow cytometry, surface levels of CXCR4 measured by mean fluorescence intensity (MFI) ratio were significantly higher in pancreatic NET cell lines (BON1, CM, QGP1) as compared with H727 and CNDT 2.5 cells (*p* = 0.01; Table 1). Membrane expression of CXCR4 occurred in > 25% of BON1 and QGP1 cells, whereas lower values were detected in CM, H727 and CNDT 2.5 cells. Following Bonferroni's post-test, the rate of expression of CXCR4 was significantly higher in BON1 and QGP1 cell lines only when compared with CNDT 2.5 cells (*p* < 0.01). Lymphocytes used as positive control showed a MFI ratio of 1.19, with 45% of positive cells. CXCL12 secretion by NET cells is summarized in Table 1, that shows how cell lines expressing low levels of surface CXCR4, namely H727 and CNDT 2.5, produced significantly higher amounts of the cytokine (*p* = 0.04). Based on these findings, we indicated BON1, CM and QGP1 as CXCR4^high^/CXCL12^low^ cell lines, whereas H727 and CNDT 2.5 cells were classified as CXCR4^low^/CXCL12^high^.

**Table 1:** CXCR4 and CXCL12 measurement in NET cell lines

		CXCR4 surface expression			CXCL12 secretion	
	MFI ratio (mean ± SE)	p*	% of positive cells (mean ± SE)	p*	pg/ml (mean ± SE)	p*
Cell lines		0.01		< 0.01		0.04
BON1	2.78 ± 0.32		26.8 ± 2.7		12.2 ± 1	
CM	3.51 ± 0.84		11.2 ± 4.5		18.7 ± 0.6	
QGP1	2.35 ± 0.42		26.3 ± 5.3		5.5 ± 0.7	
H727	1.07 ± 0.19		14 ± 2.3		154 ± 54.1	
CNDT 2.5	1.58 ± 0.46		7 ± 1.3		200.7 ± 137.3	

^*^One-way ANOVA.

### CXCL12 is inert on NET cell proliferation

CXCL12 up to 100 ng/ml was tested by MTS assay. No significant effect was observed even after 72 hrs of incubation, irrespective of the concentration of CXCL12. The time-dependent response of NET cells to 100 ng/ml of CXCL12 is depicted in Supplementary Figure 1.

### The *in vitro* osteotropism of NET cell lines is influenced by CXCL12

**Figure 1:**
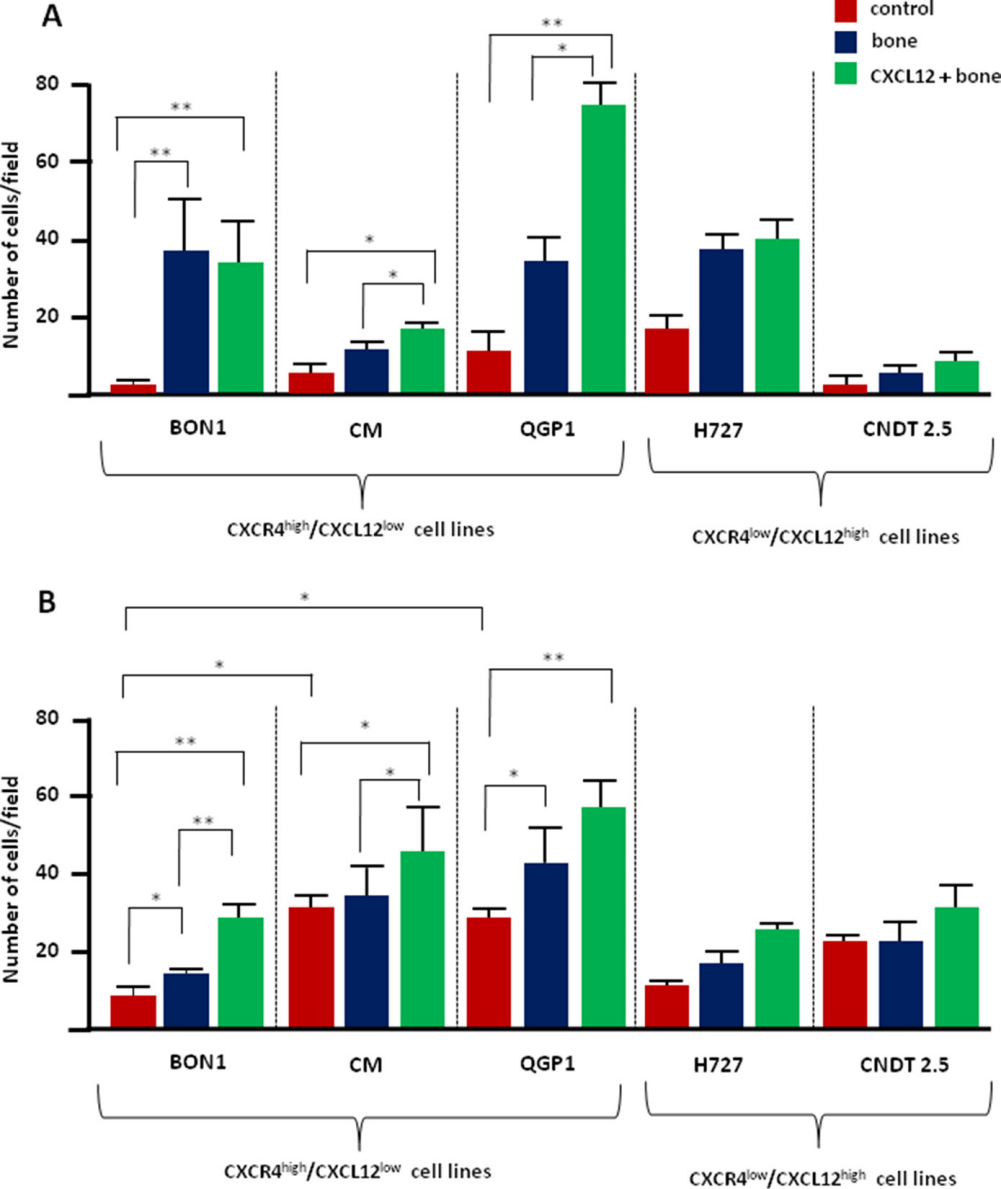
CXCL12 modulates the *in vitro* osteotropism of CXCR4^high^/CXCL12^low^ NET cell lines (**A**) The migratory potential of NET cells was measured by transwell assays. When exposed to the bone-conditioned medium, only BON1 cells significantly enhanced their migratory properties as compared with control preparations. After 2 hours of incubation with CXCL12 at 100 ng/ml, both CM and QGP1 cell lines acquired significant migratory ability towards the bone. By contrast, CXCR4^low^/CXCL12^high^ H727 and CNDT 2.5 cell lines were not significantly chemoattracted to the bone, even following CXCL12 treatment. (**B**) Exposure to bone fragments significantly increased the invasive potential of both BON1 and QGP1 cell lines, as determined by Matrigel assay. Invasiveness of the CXCR4^high^/CXCL12^low^ cell lines was further enhanced by CXCL12 pretreatment. Data are expressed as mean ± SD, and were calculated on at least three different experiments. Statistical significance is indicated by *(*p* < 0.05), or **(*p* < 0.01).

The effect of CXCL12 on both the migratory and invasive potential of NET cell lines was assessed by transwell assays. As represented in Figure 1A, NET cells showed similarly low migration towards the FCS-deprived medium (*p* > 0.05). Only BON1 cells significantly migrated in the presence of bone fragments (*p* < 0.0001), thus implying intrinsic osteotropism. This constitutive activity remained unchanged after CXCL12 pretreatment which, however, significantly improved the migration of CM and QGP1 cells towards the bone-conditioned medium (*p* = 0.02 and *p* = 0.03, respectively). On the contrary, both H727 and CNDT 2.5 cell lines failed to show osteotropism *in vitro*, and their migratory capability was not influenced by pre-conditioning with CXCL12.

We then used matrigel-coated transwell inserts to evaluate the invasive potential of NET cells (Figure 1B). Invasiveness of CM and QGP1 cell lines was natively higher than BON1 cells (*p* = 0.002) and exposure to the bone-conditioned medium further increased this activity in both BON1 and QGP1 cell lines (*p* = 0.04 and *p* = 0.03, respectively). Furthermore, pretreatment with CXCL12 enhanced the invasive potential of BON1 and CM cells (*p* = 0.004 and *p* = 0.04, respectively), while leading to a borderline increase in QGP1 cells (*p* = 0.07). As in migration experiments, H727 and CNDT 2.5 cell lines did not show any significant bone tropism, even after pretreatment with CXCL12. Taken together, these data indicate that the CXCR4^high^/CXCL12^low^ BON1, CM and QGP1 cell lines express variable levels of osteotropism *in vitro*, which is further increased by CXCR4 stimulation. On the contrary, both H727 and CNDT 2.5 cell lines, as models of CXCR4^low^/CXCL12^high^ cells, appear defective in intrinsic osteotropism and are insensitive to CXCL12.

### CXCL12 drives EMT in CXCR4^high^/CXCL12^low^ NET cells

**Figure 2:**
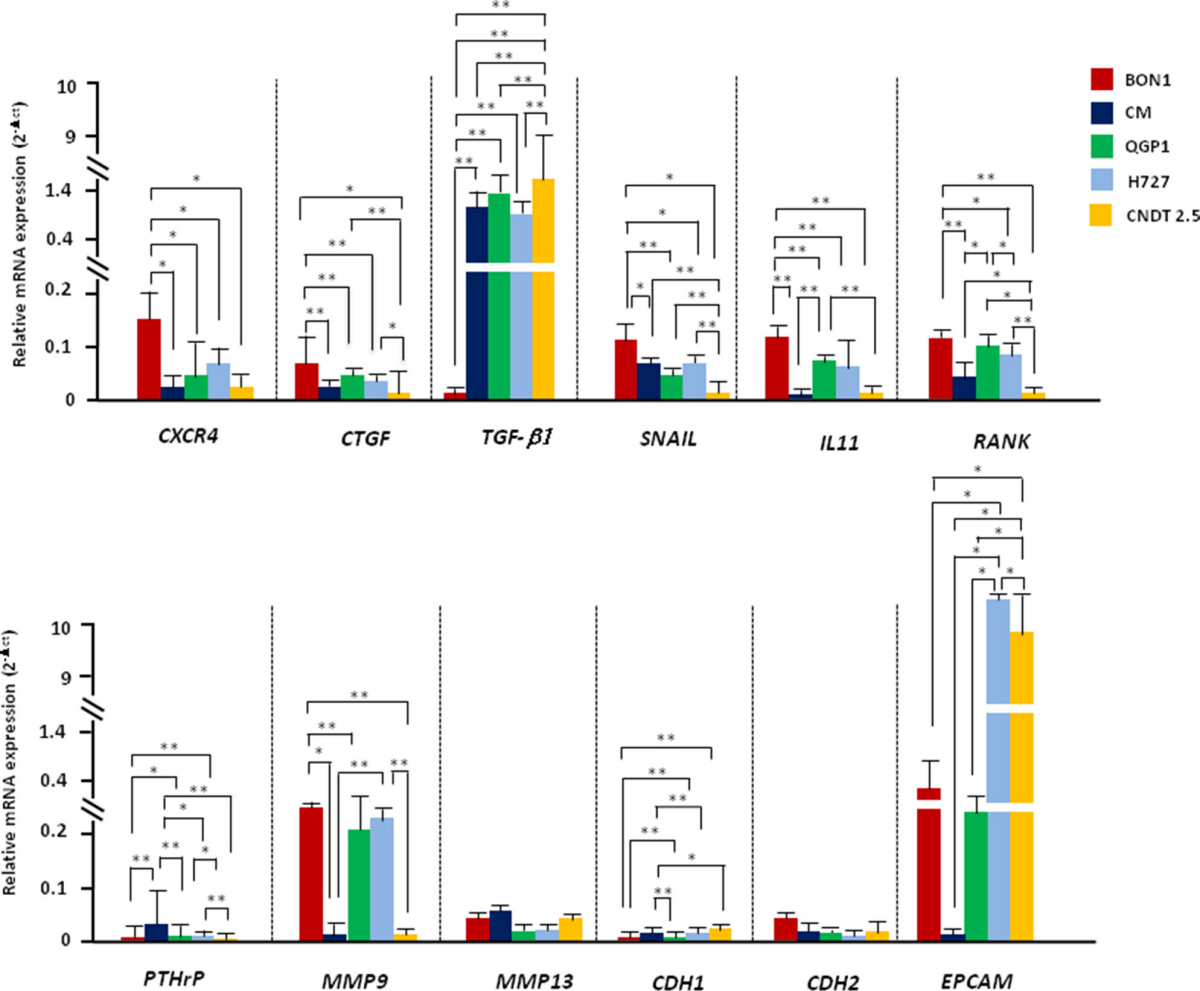
NET cell lines differ in their baseline EMT-related transcriptional profile The EMT-related transcriptional profile of NET cell lines was explored by RT-PCR. The transcriptional signature of partial EMT was more pronounced in BON1 cells, but was also present in CM and QGP1 cell lines. H727 and CNDT 2.5 cells showed prevalent epithelial features (i.e., EpCAM upregulation). Data are expressed as 2^−Δct^, using β-actin as housekeeping gene. Mean values ± SD are represented, and significant differences are marked by *(*p* < 0.05), or **(*p* < 0.01).

Since CXCL12 influenced the migration and invasiveness of several NET cell lines, we verified its potential in eliciting the transcriptional machinery driving EMT. To this aim, we screened by RT-PCR 12 EMT-related genes in NET cell lines at baseline as well as after 2 or 24 hr-treatment with CXCL12 or TGF-β1. As depicted in Figure 2, NET cell lines constitutively differed in EMT gene expression. BON1 cells, indeed, showed significantly higher mRNA of *CXCR4* (*p* = 0.04), *CTGF* (*p* < 0.001), *SNAIL* (*p* < 0.001) and *IL-11* (*p* < 0.001), in parallel with the lowest expression of *TGF-β1* (*p* < 0.0001). *RANK* was significantly upregulated in BON1 and QGP1 cells as compared with the other cell lines (*p* < 0.0001). On the other hand, *PTHrP* was minimally expressed in CM cells while virtually absent in the other cells (*p* < 0.001). There was no difference in the expression of *MMP13*, while *MMP9* mRNA was significantly higher in BON1, QGP1 and H727 cells with respect to CM and CNDT 2.5 cells. The E-cadherin encoding gene *CDH1* was overexpressed in QGP1, H727 and CNDT 2.5 cell lines rather than in BON1 and CM cells (*p* < 0.001), whereas no difference was observed in mRNA levels of *CDH2*. Finally, *EpCAM* as marker of epithelial differentiation was dramatically increased in H727 and CNDT 2.5 cells as compared with the three pNET cell lines (*p* = 0.0007). Overall, at baseline, epithelial features were prevalent in CXCR4^low^/CXCL12^high^ cell lines, whereas a more mesenchymal phenotype was observed in CXCR4^high^/CXCL12^low^ NET cell lines, particularly in BON1 cells.

**Figure 3:**
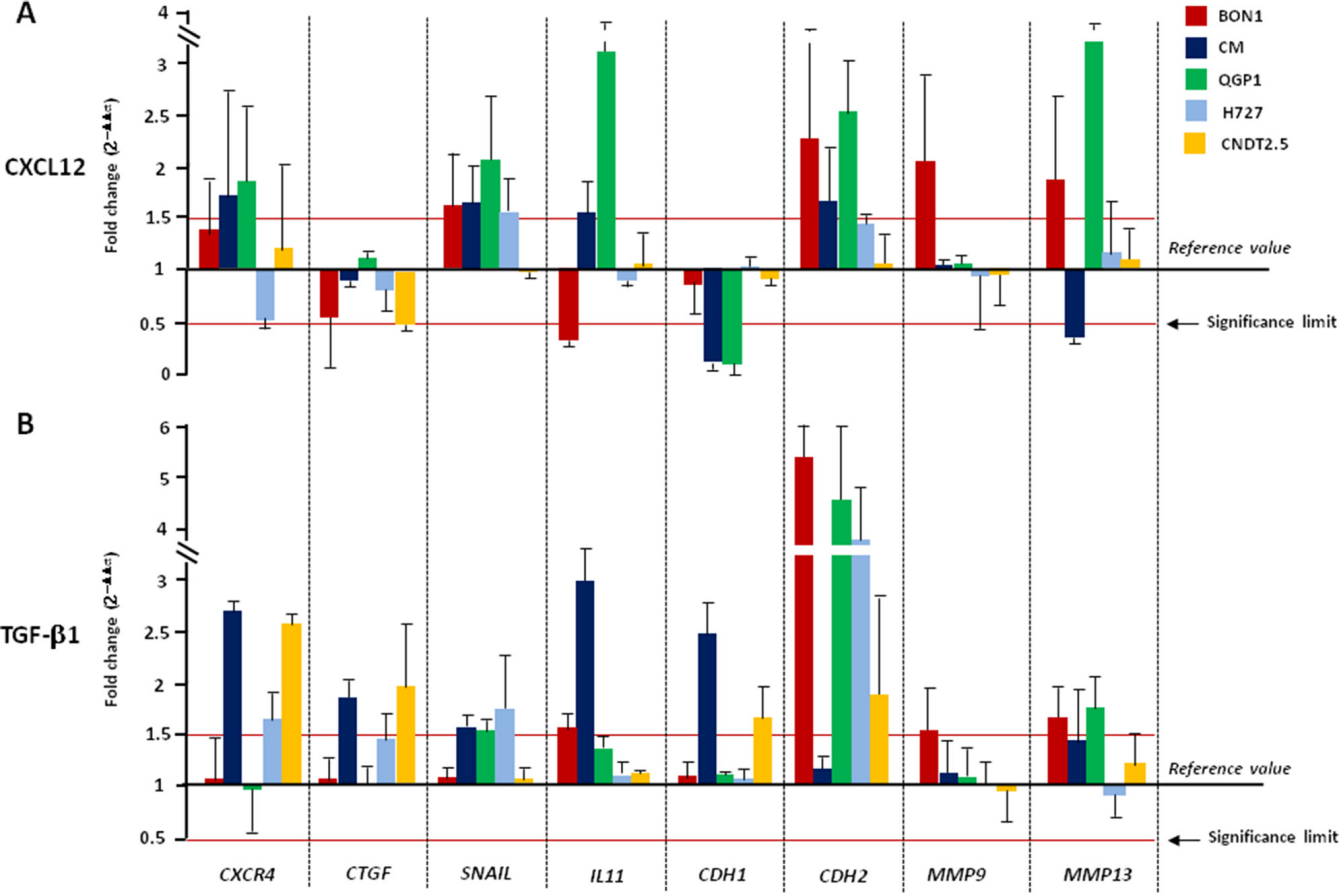
CXCL12 and TGF-β1 activate EMT in CXCR4^high^/CXCL12^low^ NET cell lines (**A**) Incubation for 2 hours with CXCL12 at 100 ng/ml variably altered the EMT transcriptional profile of CXCR4^high^/CXCL12^low^ NET cell lines, leading to cadherin switch and SNAIL upregulation. Such EMT profile was particularly marked in CM and QGP1 cells, whereas CXCL12 failed to induce significant effects on CXCR4^low^/CXCL12^high^ NET cell lines. (**B**) TGF-β1 induced EMT-related genes in both CXCR4^high^/CXCL12^low^ and CXCR4^low^/CXCL12^high^ NET cell lines. Transcript level modifications after cytokine treatment are expressed by 2^−ΔΔCT^ method using β-actin as housekeeping gene. Data are presented as mean ± SD. Statistical significance is marked by red lines.

Changes in mRNA transcription following 2 or 24 hr-treatment with CXCL12 are summarized in Figure 3A and Supplementary Figure 2A respectively. Stimulation of CXCR4 induced a complete cadherin switch in CM and QGP1 cell lines, whereas in BON1 cells upregulated *CDH2* with no effects on *CDH1* mRNA levels. Expression of *SNAIL* was also significantly increased in both BON1, CM and QGP1 cells after CXCL12 treatment. A similar upregulation was noted for *CXCR4* and *IL-11* in CM and QGP1 cell lines. *MMP9* and *MMP13* mRNAs were significantly augmented by CXCL12 in BON1 cells, and in both BON1 and QGP1 cells, respectively. In CNDT 2.5 cells, CXCL12 reduced the *CTGF* expression. The transcriptional modifications promoted by CXCL12 in NET cell lines were similar to those caused by TGF-β1, used as positive modulator of EMT [6, 14, 15] (Figure 3B). In particular, changes in the mRNA levels of *CDH2*, *SNAIL*, *IL-11*, *MMP9* and *MMP13* were comparable in either CXCL12- or TGF-β1-treated cells. Of note, downregulation of *CDH1* as crucial event in EMT was more pronounced after CXCL12 rather than TGF-β1 treatment. The transcription of *TGF-β1*, *RANK*, *PTHrP*, and *EpCAM* was neither affected by CXCL12, nor by TGF-β1 (Supplementary Figure 3). Overall, the changes seen after a 2 hr-incubation with CXCL12 or TGF-β1 persisted even after treatment for 24 hr (Supplementary Figure 2). Taken together, these data demonstrate that both CXCL12 and TGF-β1 are able to induce an EMT-like transcriptional shift in NET cell lines. However, while the effects of TGF-β1 treatment were visible in all cell lines, only CXCR4^high^/CXCL12^low^ NET cells were influenced by CXCL12 in their transcriptional profile.

### CXCR4^high^/CXCL12^low^ NET cell lines acquire a mesenchymal phenotype in response to CXCL12

**Figure 4:**
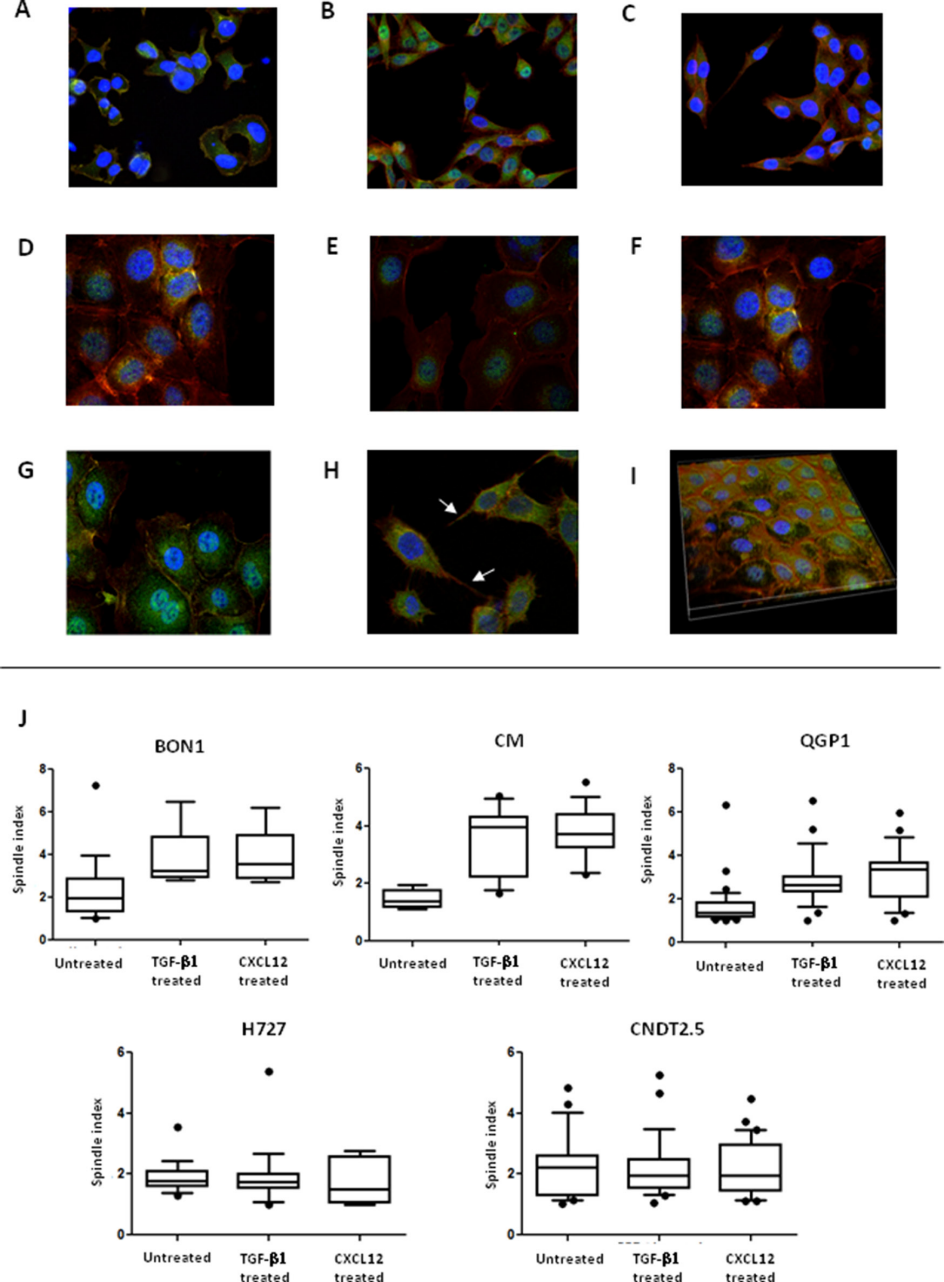
CXCL12 induces modifications in NET cell morphology Changes in NET cell morphology upon CXCL12 or TGF-β1 stimulation were assessed by confocal microscopy. At baseline, both BON1 (**A**) and H727 cells (**D**) showed a round or irregularly polygonal shape. After either CXCL12 (**B**) or TGF-β1 treatment (**C**), BON1 cells as CXCR4^high^/CXCL12^low^ cells, acquired a mesenchymal shape. By contrast, the morphology of H727 cells, representative of CXCR4^low^/CXCL12^high^ cell lines, remained unchanged after either CXCL12 (**E**) or TGF-β1 (**F**). In QGP1 cells (**H**), CXCL12 induced the formation of pseudopodia (white arrows), which were not present in control preparations (**G**). A 3D reconstruction of QGP1 cells demonstrates the subcellular localization of non-phosphorylated CXCR4 (FITC, green) in both the cytomembranous and nuclear compartments (**I**). Nuclei are stained in blue (DAPI), whereas cytoskeletal F-actin is stained in red (phalloidin). Following CXCL12 or TGF-β1 treatment, there was a significant increase of the spindle index in BON1, CM and QGP1 cells (**J**). Mean, 95% confidence interval and outliers are depicted by box and whisker plots.

We investigated the effects of CXCL12 on the shape of NET cells. As depicted in Figure 4A and 4D, before treatment both CXCR4^high^/CXCL12^low^ and CXCR4^low^/CXCL12^high^ NET cell lines displayed a polygonal or round morphology with an high nuclear-to-cytoplasmic ratio and features of anaplasia. After treatment with CXCL12, an increased proportion of spindle shaped cells was detected in BON1, CM and QGP1 cell lines (Figure 4B), but not for H727 and CNDT 2.5 cell lines (Figure 4E). Notably, cytoplasmic pseudopodia were particularly evident in both CM and QGP1 cell lines (Figure 4H). These shape variations appeared similar to those induced by TGF-β1 in both CXCR4^high^/CXCL12^low^ and CXCR4^low^/CXCL12^high^ cells (Figure 4C, 4F). As shown in Figure 4J, in BON1, CM and QGP1 cells the spindle index [16] significantly increased following CXCL12 treatment (*p* = 0.04, *p* < 0.0001 and *p* < 0.0001, respectively). Similar effects were seen after TGF-β1 treatment.

### CXCR4 accumulates within the nuclei of CXCR4^high^/CXCL12^low^ NET cells undergoing EMT

**Figure 5:**
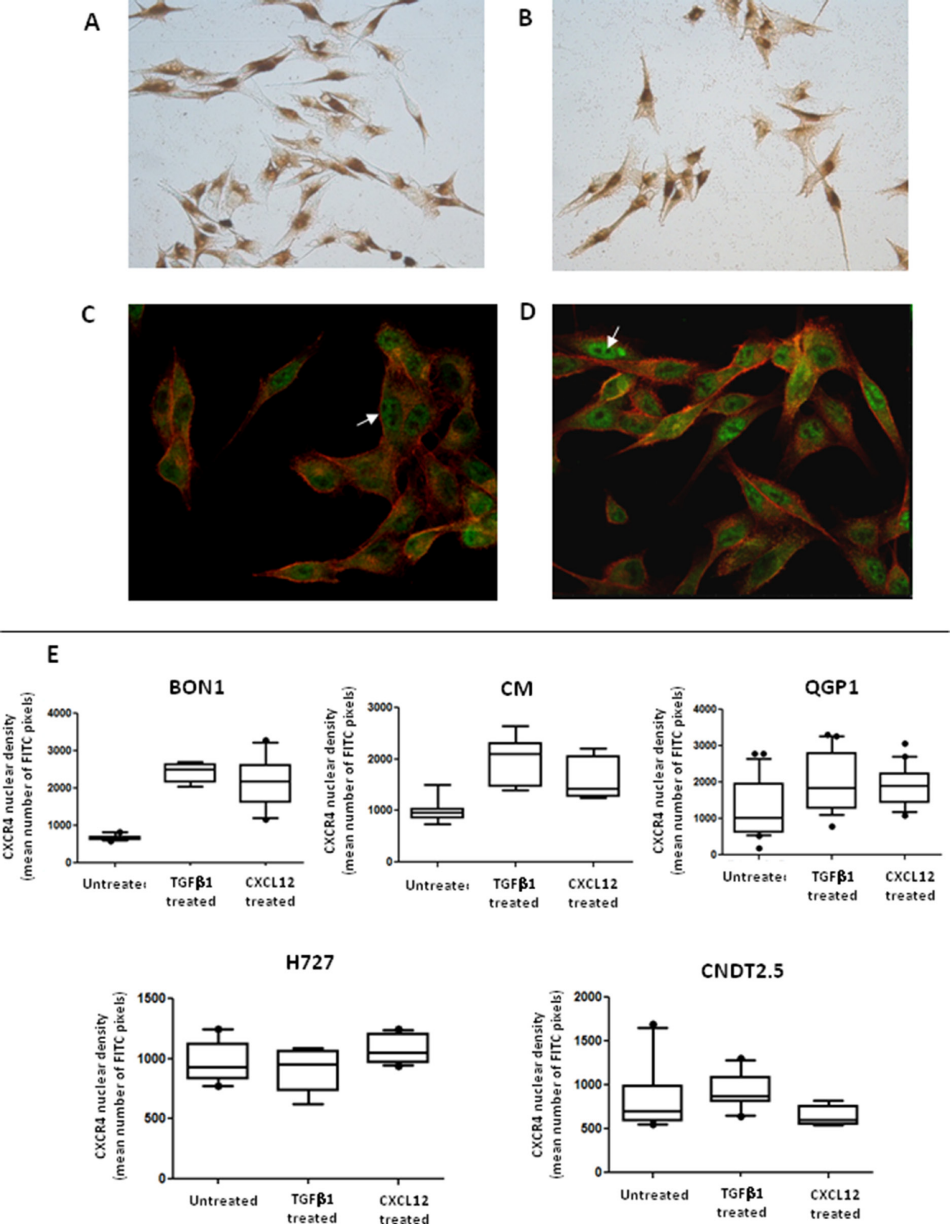
Activation of EMT is associated with CXCR4 nuclear accumulation The nuclear localization of CXCR4 in NET cell lines was revealed by ICC, as represented here in CM cells. Both a polyclonal (**A**) or monoclonal (**B**) Abs were used, resulting in minor staining differences. The nuclear accumulation of non-phosphorylated CXCR4 molecules (white arrows) was also detected by confocal microscopy (**C**). CXCL12 treatment (similarly to TGF-β1 treatment, not shown) led to a significant increase of the CXCR4-related FITC nuclear intensity (**D**). These data were confirmed by computational analysis of the CXCR4 nuclear density. As represented by box and whisker plots, the mean number of FITC pixels emitted within the nuclear area significantly increased following incubation with CXCL12 in CXCR4^high^/CXCL12^low^ NET cell lines (BON1, CM, QGP1).

We have previously reported a positive nuclear staining in FFPE NET samples subjected to immunohistochemistry (IHC) using a polyclonal Ab against CXCR4 [3]. When investigated by ICC with the same Ab, all NET cell lines showed the nuclear localization of the receptor in the presence of a weak membranous labeling, as represented for CM cells in Figure 5A. As shown in Figure 5B, additional ICC experiments employing the mAb UMB-2, which specifically binds CXCR4 when its C-terminal epitope is non-phosphorylated as result of ligand stimulation, revealed a diffuse membranous and cytoplasmic staining, in the presence also of nuclear immunoreactivity. By confocal microscopy, the UMB-2 mAb demonstrated in all 5 cell lines an ubiquitous expression of non-phosphorylated CXCR4 throughout the cell, with distinct immunofluorescent foci at the plasma membrane, within the cytoplasm and particularly around or within the nuclear compartment, as showed by the co-localization with the DAPI labeling (Figure 4). Although diffusely positive within the cell nuclei, CXCR4 was absent in nucleoli (Figure 5C).

To explore possible interconnections between the subcellular localization of non-phosphorylated, thus functionally bioavailable CXCR4 and the occurrence of EMT in NET cell lines, both confocal microscopy and western blot analysis after biochemical fractionation of nuclear and extra-nuclear components were also used. In CXCL12- or TGF-β1-treated NET cells with CXCR4^high^/CXCL12^low^ phenotype, we noted a striking increase of the CXCR4-related FITC fluorescence in cell nuclei (Figure 5C–5D). This finding was further confirmed by computational analysis of the nuclear density of CXCR4 using the NIS software. In fact, as depicted in Figure 5E, the mean number of pixels emitted by the FITC fluorochrome in the nuclear area were significantly increased in BON1 (*p* < 0.0001), CM (*p* = 0.0004) and QGP1 cells (*p* = 0.006) by stimulation with either CXCL12 or TGF-β1, while remained unchanged in H727 and CNDT 2.5 cell lines. These data suggest that an apparent nuclear translocation of ligand-actionable CXCR4 occurs in NET cells exposed to EMT-inducing stimuli as provided by CXCL12 or TGF-β1.

**Figure 6:**
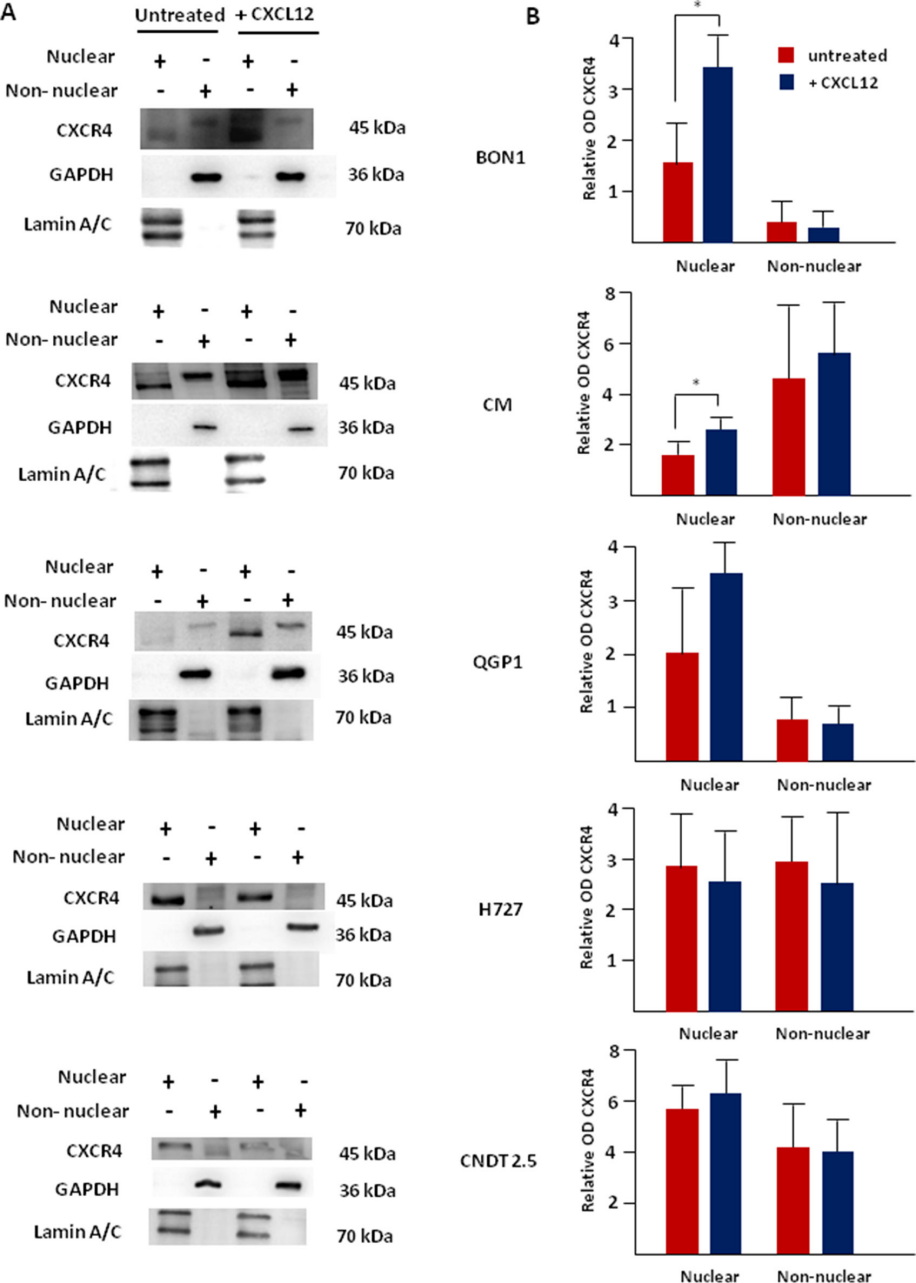
Nuclear accumulation of CXCR4 following its agonist stimulation is predominant in CXCR4^high^/CXCL12^low^ NET cell lines After biochemical fractionation, nuclear and non-nuclear aliquots of NET cells were probed with the anti-CXCR4 mAb UMB-2, which recognizes the receptor only when inactive and non-phosphorylated. Western blot showed a significant upregulation of the nuclear expression of the actionable fraction of CXCR4 following CXCL12 incubation in both BON1 and CM cell lines. In QGP1 cells, the upregulation of nuclear CXCR4 approached statistical significance (*p* = 0.08). No substantial modifications were seen in CXCR4^low^/CXCL12^high^ NET cell lines treated with CXCL12. Interestingly, the non-nuclear expression of non-phosphorylated CXCR4 was not significantly influenced by CXCL12 in all cell lines. Lamin A/C and GAPDH were internal controls in both nuclear and non-nuclear preparations, respectively.

To further verify our data, western blot experiments were completed after biochemical fractionation of NET cell lines in nuclear and non-nuclear aliquots. As shown in Figure 6A, the monomeric isoform of non-phosphorylated CXCR4 (45 kDa) was dually expressed in both nuclear and non-nuclear fractions in all cell lines. In H727 and CNDT 2.5 cells, no effect was detected following CXCL12 treatment in terms of CXCR4 expression (Figure 6B). On the other hand, CXCL12 induced a significant overexpression of the nuclear fraction of non-phosphorylated CXCR4 in BON1 and CM cells (*p* = 0.01 and *p* = 0.04, respectively). In QGP1 cell lines the upregulation of nuclear CXCR4 approached statistical significance (*p* = 0.08). Of interest, in BON1 cells, the majority of unbound CXCR4 was compartmentalized within the nuclei both at baseline and after CXCL12 treatment. The purity of the subcellular aliquots extracted from each cell line was confirmed by detection of lamin A/C and GAPDH in the nuclear and extra-nuclear fractions, thus excluding that the presence of CXCR4 in the nuclei was due to non-nuclear contamination. There was an excellent degree of correlation between the levels of nuclear CXCR4/Lamin A by western blot, and the intensity of nuclear fluorescence emitted by CXCR4, as assessed by computational analysis of confocal microscopy images (*r* = 0.87; *p* = 0.03).

### CXCR4 silencing disables EMT occurrence in NET cell lines

**Figure 7:**
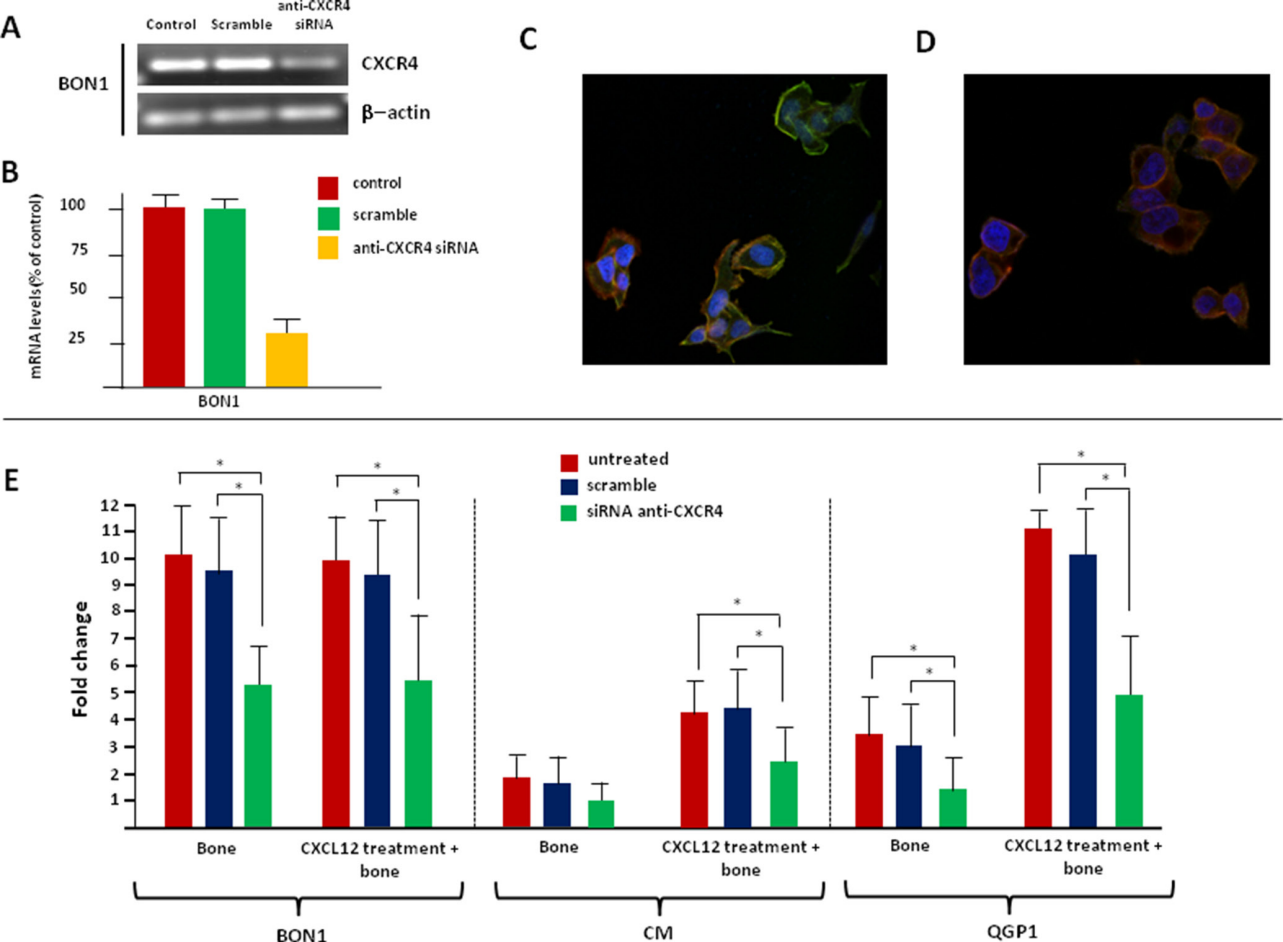
Silencing of CXCR4 reverts the EMT-like changes induced by CXCL12 in NET cells The transfection efficiency of the anti-CXCR4 siRNA was evaluated by qualitative PCR (**A**), RT-PCR (**B**) and confocal microscopy (**C**). Overall, siRNA treatment induced a reduction of CXCR4 mRNA up to 70% in BON1 cells, as compared with original control values. A similar decrease was observed in terms of protein expression in the same cell line. (**D**) After incubation with CXCL12, siRNA-treated BON1 cells maintained a polygonal shape. (**E**) Silencing of CXCR4 reduced the migratory potential of CXCR4^high^/CXCL12^low^ NET cells, even following CXCL12 treatment.

To verify the functional relevance of CXCR4 in supporting EMT in CXCR4^high^/CXCL12^low^ NET cell lines, CXCR4 loss-of-function studies were carried out. Receptor knockdown by siRNA treatment was confirmed by qualitative PCR, RT-PCR, and confocal microscopy. Figure 7A–7C and Supplementary Figure 4A show that both mRNA and protein levels of CXCR4 were dramatically reduced in siRNA-treated NET cells, but not in mock- or scramble-treated cells. Remarkably, very weak-to-no membrane and nuclear immunolabeling was detectable in CXCR4-silenced cells, thus confirming the specificity of the UMB-2 mAb.

Changes in intrinsic and CXCL12-enhanced bone tropism of NET cell lines were evaluated before and after siRNA transfection, as summarized in Figure 7E. CXCR4 knockdown significantly reduced the migration towards the bone in BON1 and QGP1 cells. Moreover, after CXCR4 silencing, CXCL12 was unable to upregulate the *in vitro* bone tropism of both BON1, CM and QGP1 cells. Similarly, the invasive potential of CXCR4^high^/CXCL12^low^ NET cell lines was not altered by CXCL12 following anti-CXCR4 siRNA transfection (Supplementary Figure 4B), thus suggesting that the previously documented pro-migratory and pro-invasive effects of this cytokine were specifically mediated by CXCR4 itself.

To further confirm the role of CXCR4 as critical regulator of EMT in NET cell lines, we assessed the EMT-related transcriptional profile of CXCR4-silenced cells after 2 hr-CXCL12 stimulation. As expected, in CXCR4-silenced cells CXCL12 was unable to induce substantial modifications in the expression of EMT-related genes (*CDH1*, *CDH2*, *EPCAM*, *CXCR4*, *TGF-β1*, *SNAIL*, *CTGF*, *IL11*, *PTHrP*, *RANK*, *MMP9* and *MMP13*), as proven by mRNA levels that were, indeed, comparable with those of untreated, mock- and scramble-treated NET cells (data not shown). Consistently with these observations, we also found that silencing of CXCR4 inhibited the acquisition of a mesenchymal phenotype in response to CXCL12. In fact, no changes in the shape of siRNA-transfected BON1, CM, and QGP1 cells were observed following the stimulation with the chemokine as compared with untreated control cells. Representative experiments with BON1 cells are shown in Figure 7C–7D. Analysis of the spindle index showed no difference between control cells and CXCL12-treated NET cells after CXCR4 deprivation (Supplementary Figure 5).

Taken together, these findings provide a definite evidence that the EMT-promoting signals conveyed by CXCL12 are critically sensed by CXCR4 which regulates transcriptional, conformational and functional modifications that promote the osteotropism in NET cell lines.

## DISCUSSION

This study defines the role of the CXCL12/CXCR4 axis in the biology of bone-colonizing NETs, at least *in vitro*. It demonstrates that the EMT-promoting signals conveyed by CXCL12 to NET cells are critically sensed by surface CXCR4 leading to transcriptional, structural and functional modifications that culminate in enhanced tumor osteotropism. Moreover, it shows that the bioavailable fraction of CXCR4 accumulates within the nuclei of EMT-undergoing NET cells, thus suggesting that unique functions of the receptor may segregate according to its subcellular distribution.

NETs are curable only when localized, but up to 95% of patients are diagnosed at stage IV [17]. In most recent phase III trials of advanced NETs, the skeleton is a frequent site of metastases [18, 19], and the association between NET bone colonization and poor patient outcome is consistently reported by retrospective studies [4, 5]. Based on these premises, osteotropic NETs can be seen as aggressive subtypes of these tumors and the identification of their molecular hallmarks may be helpful in the prevention and development of new therapies.

In the present study, we investigated the CXCL12/CXCR4 axis in multiple cell lines representative of pancreatic, ileal and bronchial NETs. We found that the pancreatic BON1, CM and QGP1 NET cell lines expressed membrane CXCR4 at high density, but only in a relatively small percentage of cells, namely up to 27%. In spite of an overlapping percentage of positivity, other NET cells as H727 and CNDT 2.5, displayed a three-fold lower density of CXCR4. However, an inverse correlation between CXCR4 expression and CXCL12 secretion was also observed in our work, that dichotomically categorized BON1, CM and QGP1 as CXCR4^high^/CXCL12^low^ NET cell lines and H727 and CNDT 2.5 as CXCR4^low^/CXCL12^high^ cells. In this context, an autocrine regulatory loop between CXCL12 and CXCR4 can be hypothesized also for NETs, since evidence in other cancers suggests that persistent ligand stimulation causes CXCR4 degradation, as well as epigenetic regulation of the entire molecular synapse [7, 20, 21]. The putative association between CXCR4 overexpression, EMT activation and increased risk of NET bone metastases [3] led us to hypothesize a selection process whereby the activation of the CXCL12/CXCR4 axis skews NET cells toward a preponderance of EMT-upregulated clones. To test this hypothesis, we first ruled out the proliferation effects of CXCL12 on NET cell lines, since an accelerated proliferation could justify *per se* a more aggressive tumor behavior, thus promoting higher rates of skeleton colonization. In our hands, CXCL12 was inert on NET cell proliferation, in line with work from others [9].

According to the “metastasis seed pre-selection” hypothesis [22], the tumor spread to distant organs is related to peculiar gene signatures of the primary tumors. Therefore, we investigated the effects of CXCL12 on the *in vitro* osteotropism of NET cells, and correlated the migratory changes with transcriptional modifications induced by agonist stimulation of CXCR4. Notably, only CXCR4^high^/CXCL12^low^ pNET cell lines BON1 and QGP1 displayed intrinsic migratory and invasive potential towards the bone, in line with the increased osteotropism observed in patients with pNETs. Indeed, significantly higher odds of bone colonization have been recently shown for pNETs as compared with all other NET primaries [23]. In CXCR4^low^/CXCL12^high^ NET cell lines, autocrine stimulation by CXCL12 probably inhibited cell homing to the bone as result of an inadequate gradient effect. These findings appear in line with other studies correlating the CXCR4 upregulation/CXCL12 downregulation in tumor cells with their enhanced spreading to CXCL12-overexpressing sites [13, 24]. Intriguingly, when evaluated in their baseline transcriptional profile, CXCR4^high^/CXCL12^low^ NET cell lines show the signature of partial EMT [6], namely low levels of both *CDH1* and *EpCAM* expression. In this context, the association between functional and transcriptional features of BON1 cells was paradigmatic. In fact, while being the only cell line with both migratory and invasive intrinsic potential, BON1 showed a profile characterized by high *CXCR4* and *CTGF* transcription in the presence of downregulated *TGF-β1*. This specific EMT signature was described in our previous work as predictive of NET bone colonization [3].

Agonist stimulation of CXCR4 significantly increased the migratory/invasive capabilities of CXCR4^high^/CXCL12^low^ NET cell lines, whereas no effect was detected on CXCR4^low^/CXCL12^high^ NET cells. This finding depicts several aspects of the CXCL12/CXCR4 biology in these tumors. First, CXCL12 is not only involved in migration of NET cells, but is also able to enhance their sensitivity to the complex cytokine milieu of the bone marrow. Second, transient administration of exogenous CXCL12 or chronic production of endogenous CXCL12 may apparently have opposite effects on NET cell migration and invasiveness. This effect has been already reported in colorectal cancer [25].

Changes of the *in vitro* osteotropism of NET cell lines following CXCL12 incubation were paralleled by concomitant modifications in the EMT-related transcriptional profile. Indeed, while being inactive in CXCR4^low^/CXCL12^high^ NET cell lines, CXCR4 agonist stimulation caused an EMT-like transcriptional shift in the CXCR4^high^/CXCL12^low^ counterpart. This was particularly evident in CM and QGP1 cells, in which CXCL12 promoted a complete cadherin switch along with *SNAIL*, *CXCR4* and *IL-11* overexpression. In the BON1 cell line, bearing remarkable signs of partial EMT at baseline, the CXCL12-induced transcriptional modifications were less evident, although present and still able to increase the cellular invasiveness.

To further support our hypothesis that CXCL12 elicits EMT in specific subsets of NET cells, we compared the transcriptional changes induced by CXCL12 or TGF-β1 in cell lines. TGF-β1 can act as either promoter, via EMT activation, or suppressor of tumor progression, depending on the degree of tumor malignancy [26]. In this context [3], we have reported its protective role in low-to-intermediate grade carcinoids, but the higher malignancy of NET cell lines is well documented [27, 28]. Therefore, here we used TGF-β1 as positive regulator of EMT and observed that this cytokine induced the transcriptional signature of EMT in all NET cell lines, with modifications similar to those caused by CXCL12 in CXCR4^high^/CXCL12^low^ NET cells.

When evaluated in their morphology upon stimulation with CXCL12 or TGF-β1, NET cells underwent similar changes. Both cytokines, indeed, prompted CXCR4^high^/CXCL12^low^ NET cells to acquire a mesenchymal shape including the presence of pseudopodia, whereas no significant changes were revealed in H727 and CNDT 2.5 cells. Why CXCR4^low^/CXCL12^high^ NET cells treated with TGF-β1 remained morphologically unchanged during EMT activation is unclear. However, subliminal stimulation of the EMT-activating machinery or lack of other convergent stimuli for the full EMT activation can be hypothesized.

To ascertain that the EMT-promoting signals conveyed by CXCL12 were specifically sensed by CXCR4 in NET cell lines, we carried out loss-of-function studies via siRNA silencing. Knockdown of CXCR4 was able to revert the CXCL12-induced EMT phenotype in CXCR4^high^/CXCL12^low^ NET cell lines. In particular, both transcriptional, conformational and pro-migratory effects of CXCL12 were silenced in cells lacking the expression of CXCR4, thus supporting its crucial role in mediating the effects of CXCL12. Although CXCR4 is a membrane receptor, its intracellular segregation following CXCL12 stimulation has been widely reported and associated with high tumor malignancy [7, 29–32]. We have previously documented a positive nuclear staining in primary NETs by IHC using a polyclonal anti-CXCR4 Ab [3], whereas a purely membranous expression of the receptor was described by Kaemmerer *et al*. in both lung and gastroenteropancreatic NETs employing the anti-CXCR4 mAb UMB-2 [10, 11]. Here, we investigated the subcellular expression of CXCR4 in NET cell lines by using both Abs Although in the presence of a more defined cytomembranous staining with UMB-2, both Abs definitely showed nuclear immunoreactivity for CXCR4. Both the relatively low concentration of the mAb used (1:200, as compared with 1:100 in [30]) and the absence of immunoreactivity in CXCR4-silenced cells supported the specificity of our findings.

Ligand stimulation of CXCR4 induces phosphorylation of several serine residues in the C-terminal domain of the receptor, thus leading to stabilization of the interaction with β-arrestin and receptor internalization [21, 33, 34]. Evidence demonstrates that the UMB-2 mAb is phosphosensitive, and binds CXCR4 only when the C-terminal epitope is non-phosphorylated and the receptor consequently inactive [33]. Here, we observed accumulation of the non-phosphorylated fraction of CXCR4 in the nuclei of CXCR4^high^/CXCL12^low^ NET cell lines following incubation with CXCL12 or TGF-β1. Although the origin and functional relevance of nuclear CXCR4, as well as the mechanisms enrolled in its translocation are undefined, the direct involvement of functionally bioavailable CXCR4 in the transcriptional activation of EMT cannot be excluded. Future research is thus needed to characterize the nuclear activity of CXCR4 as part of the transcriptional machinery driving the EMT process, and/or as modulator of the secondary G-protein coupled receptor (GPCR) signals that regulate the EMT at the nuclear level. Interaction between the nuclear pool of both CXCR4 and protein G_αi_ has been already documented in prostate cancer [35], and supports the hypothesis that CXCR4 may regulate the signaling from inside the nucleus.

All the investigations described in the present work have been carried out using immortalized cell lines. Since the genomic and biologic resemblance of NET cell lines and their primary tumor counterparts has been repeatedly questioned [27, 36–38], extrapolation of our findings to the clinical scenario should be performed very carefully. In this context, as skepticism has been raised particularly regarding the authenticity of the neuroendocrine background of CNDT 2.5 cells [39], findings related to this cell line, representative of midgut carcinoids or of non-NET tumors expressing CXCR4 at low levels, should be interpreted with particular caution.

Although bearing the limitations of an *in vitro* only study, this work shed lights on several mechanisms by which NET cells colonize the skeleton, and paves the way to new therapies for these tumors. Since CXCR4 agonism seems to act as a robust promoter of EMT in NETs, CXCR4 antagonism may be of therapeutic interest. Prior work has demonstrated that direct blockade of CXCR4 by AMD3100 exerts antiproliferative effects in NETs [9], but its anti-metastatic properties via EMT-inhibition have never been explored. In multiple myeloma, the anti-CXCR4 mAb ulocuplumab was recently shown to inhibit the malignant plasma cell dissemination by suppressing the EMT-like phenotype [40], and similar effects cannot be excluded in NETs. Given that the subcellular distribution of CXCR4 may influence its function, and that nuclear translocation of its bioavailable fraction is consistently noted in NET cells undergone EMT, nuclear import machinery modulators should be tested in their anti-EMT or anti-osteotropic properties in these malignancies.

## MATERIALS AND METHODS

### Cell lines, patient tissue and cytokines

The lung carcinoid H727 cell line was purchased from the American Type Cell Collection (ATCC, Milan, Italy) and was verified at our institution as recommended (ATCC Technical Bulletin no. 8; Manassas, ATCC; 2008). BON1, CM and QGP1 pancreatic NET cell lines were kindly provided by Dr. M. Donadelli (University of Verona, Italy), while midgut carcinoid CNDT 2.5 cells were a gift from Dr. L.E. Lee (M.D. Anderson Cancer Center, Houston, TX, USA). BON1 and CNDT 2.5 cells were cultured in DMEM F12 (Gibco, Life Technologies, Turin, Italy) complete medium. For CNDT 2.5 cells, sodium pyruvate, MEM vitamin solution and MEM nonessential amino acids (Gibco) were added, as previously described [41]. QGP1, CM and H727 cell lines were grown in 10% FCS RPMI 1640 and maintained at 37°C in a 5% CO_2_-incubator.

Healthy bone fragments of 3–5 mm^3^ including equivalent amounts of cortical/spongy bone were obtained from de-identified patients undergoing post-traumatic orthopedic surgery, according to a protocol approved by the Ethical Committee of the University of Bari. CXCL12 (R&D Systems, Minneapolis, MN, USA) was diluted in PBS, whereas TGF-β1 (PeproTech, Rocky Hill, NJ, USA) was suspended in 10 mM citric acid at pH 3.

### Assessment of CXCR4 expression and CXCL12 secretion

Flow cytometry investigated the expression of membrane CXCR4 in NET cell lines. After 2 hr of incubation with or without CXCL12 at 100 ng/ml, 1 × 10^5^ cells at ~70% confluency were mechanically detached and incubated at room temperature for 20 minutes in dark with 5 μl/100 μl of the APC-conjugated anti-human CXCR4 mAb (Affymetrix, Santa Clara, CA, USA). Human lymphocytes from healthy donors served as positive control cells, while APC-conjugated mouse IgG_2a_ (Beckman Coulter, Brea, CA, USA) were used for isotypic controls. After repeated PBS washing, the cells were analyzed by FACScanto (Becton-Dickinson, Mountain View, CA, USA) for both percentage of positive cells and MFI. MFI ratios were calculated by dividing the MFI for CXCR4 by the mean fluorescence of the respective nonspecific isotype control, as reported elsewhere [42]. Secretion of CXCL12 by NET cell lines was measured by ELISA (Abcam, Cambridge, MA, USA), as described [43].

### Proliferation assays

To test the effects of CXCL12 on NET cell proliferation, the 3-(4,5-dimethylthiazol-2yl)-5-(3-carboxymethoxyphenyl)-2-(4-sulfophenyl)-2H-tetrazolium (MTS) assay (Promega, Madison, WI, USA) was carried out [44]. Briefly, cells were incubated with CXCL12 at 25, 50, 100 ng/ml and analyzed after 24, 48 and 72 hr. The proliferation was then estimated with respect to the absorbance of untreated controls in biological triplicates.

### Migration and invasion assays

To assess the *in vitro* osteotropism of NET cell lines, we used 24-well plates including BD Falcon^TM^ cell culture 8 μm inserts, and BD BioCoat^TM^ Matrigel^TM^ invasion chambers (Becton Dickinson Bioscience, Bedford, MA, USA) for migration and invasion assays, respectively. Briefly, after overnight serum starvation, cells (10^4^/well) were seeded onto the upper chamber of the inserts, while 1% FCS RPMI 1640 was added to the lower chamber in the presence or absence of bone fragments. After 24h of incubation, cells on the upper surface of the membrane were removed with a cotton swab, while cells adhering to the underside of the insert were fixed with formaldehyde, then stained with 4′,6-diamidino-2-phenylindole dihydrochloride (DAPI; Sigma Aldrich), and finally counted in ten random fields at 40x magnification under a UV microscope (Leica DMRE, Heidelberg, Germany). NET cells pretreated with CXCL12 at 100 ng/ml for 2 hr were similarly tested in their migratory and invasive potential. Each experiment was performed in triplicate.

### EMT-related transcriptional profile

Real time (RT)-PCR was used to determine the expression levels of the EMT markers *CDH1* (coding for E-Cadherin), *CDH2* (coding for N-Cadherin), *EPCAM*, *CXCR4*, *TGF-β1*, *SNAIL*, *CTGF*, *IL11*, *PTHrP*, *RANK*, *MMP9* and *MMP13* in NET cell lines. mRNA levels were compared after logarithmic transformation of the raw C_T_ data (2^−ΔCt^) [45]. Gene transcription was then assessed after 2 or 24 hr treatment with CXCL12 at 100 ng/ml or TGF-β1 at 50 ng/ml as positive control for EMT [6, 14, 15], and compared with expression levels in relative untreated preparations using the 2^−ΔΔCt^ method [45]. Specific primers used for mRNA amplification are listed in SupplementaryTable 1. β-actin was used as housekeeping gene.

### Cell morphology and CXCR4 subcellular localization

After relative incubations, NET cells were fixed in paraformaldehyde, permeabilized by 0.1% Triton X-100 (Sigma Aldrich) and incubated overnight at 4°C with a rabbit anti-CXCR4 mAb (UMB2 clone, ab124824, Abcam) at 1:200 dilution. Cells were then incubated at room temperature with a secondary goat anti-rabbit FITC-conjugated Ab, while TRITC-conjugated Phalloidin (P1951, Sigma Aldrich) and DAPI were used to visualize the cytoplasmic and nuclear compartments, respectively. Samples were analyzed under a confocal laser scanning microscope (C2plus, Nikon Instr., Lewisville, TX, USA), and imaged in sequential scan mode. A dedicated software (NIS element software, Nikon Instr) was used for pixel count and analysis of nuclear-FITC intensity. As a measure of mesenchymal phenotype, the spindle index was calculated as ratio of maximum length to maximum width of at least 100 cells from 10 random fields observed at 40X [16].

CXCR4 was also investigated by immunocytochemistry (ICC) using either a rabbit polyclonal (ab2074, Abcam) or monoclonal Ab (UMB2 clone, Abcam). Briefly, NET cells were fixed in paraformaldehyde (Sigma Aldrich) and then incubated overnight at 4°C with the primary polyclonal (dilution: 1:100) or monoclonal (dilution: 1:200) anti-CXCR4 Ab, using human lymphocytes as positive controls. After treatment with a biotinylated anti-rabbit secondary Ab (Vector Laboratories, Burlingame, CA, USA), CXCR4 expression was revealed by the diaminobenzidine reaction and the slides were inspected under a light microscope. Immunoreactivity was scored as described [3].

### Western blot

Nuclear and cytoplasmic NET cell fractions were prepared by NE-PER extraction kit (Life Technologies) and 10 μg of cytoplasmic- or nuclear-purified protein lysates from both treated and untreated cells were investigated by Western blot with the rabbit anti-CXCR4 mAb UMB-2 (Abcam) at 1:1000 dilution. Lamin A/C and GAPDH detection was used as intra-assay control for both purity and loading of nuclear and non-nuclear fraction lysates, respectively. The density of CXCR4 bands was calculated by ImageQuantTL (GE Healthcare, Little Chalfont, UK) with respect to Lamin A/C or GAPDH in three different experiments whose values were expressed as mean ± SD.

### CXCR4 loss-of-function studies

To assess its role in EMT, CXCR4 was silenced in BON1, CM and QGP1 cells by using small interfering RNA (siRNA). Scramble probes (Ambion, Life Technologies, Carlsbad, CA, USA) were used as control. Two sense (GGCAGUCCAUGUCAUCUACtt; GGAAGCUGU UGGCUGAAAAtt) and two antisense oligonucleotides (GUAGAUGACAUGGACUGCCtt; UUUUCAGCCAA CAGCUUCCtt; Ambion) were combined for the silencing procedure [46]. Briefly, cells were seeded at 500,000/well and then transfected 4 hrs later by adding 3 μl/well of Lipofectamine 3000 Reagent (Invitrogen, Carlsbad, CA, USA) and siRNAs at a final concentration of 60 nmol/L. CXCR4-silenced cells were thus evaluated in their migratory and invasive capabilities, EMT-related transcriptional profile, and cell morphology.

### Statistical analysis

Data were analyzed using Student's *t*-test or one-way ANOVA with Bonferroni post-test, as appropriate. The Spearman test was used for correlation analysis. All tests were two-sided, and a *p*-value <0.05 was considered statistically significant. Statistical analysis was performed using GraphPad Prism 5 software (GraphPad Software, La Jolla, CA, USA).

## SUPPLEMENTARY MATERIALS FIGURES AND TABLES


