# Supplementary material for: Osteotropism of neuroendocrine tumors: role of the CXCL12/CXCR4 pathway in promoting EMT in vitro
Source: Oncotarget. 2017 Feb 6;8(14):22534–49. doi: 10.18632/oncotarget.15122 (PMC5410243; doi:10.18632/oncotarget.15122)
Supplement: Supplementary file 1 [file oncotarget-08-22534-s001.pdf]

Osteotropism of neuroendocrine tumors: role of the CXCL12/ CXCR4 pathway in promoting EMT *in vitro*

Supplementary Materials

Supplementary Table 1: Primer sequences used for RT-PCR experiments

Gene	Forward primer 5′ > 3′	Reverse primer 5′ > 3′
CTGF	GGCCTCTTCTGTGACTTCGG	CTCTGGAAGGACTCTCCGCT
CXCR4	TGGGTGGTTGTGTTCCAGTT	TTGGAGTGTGACAGCTTGGA
E-CAD	AGCAGAACTAACACACGGGG	ACCCACCTCTAAGGCCATCT
EPCAM	AGCGAGTGAGAACCTACTGGA	CGCGTTGTGATCTCCTTCTGA
IL-11	TGAGCCTGTGGCCAGATACAGC	AGGTTGTGGTCCCCGTCAGC
MMP9	ACTTTGACAGCGACAAGAAGTGGG	ATGCCATTCACGTCGTCCTTATGC
MMP13	TTGAGCTGGACTCATTGTCGGGC	TCTCGGAAGCCTCTCAGTCATGGAG
N-CAD	CCAGCCTCCAACTGGTATCTT	TCTACTGCATGTGCCCCTAA
PTHrP	GTCTCAGCCGCCGCCTCAA	GGAAGAATCGTCGCCGTAAA
RANK	ATTCTGCTTCTCTTCGCGTCTGTGG	ACTGACACAACTGTCACCTGAGGAC
SNAIL	CCAGACCCACTCAGATGTCAA	GGACTCTTGGTGCTTGTGGA
TGFβ	CGCGTGCTAATGGTGGAAAC	GTTCAGGTACCGCTTCTCGG

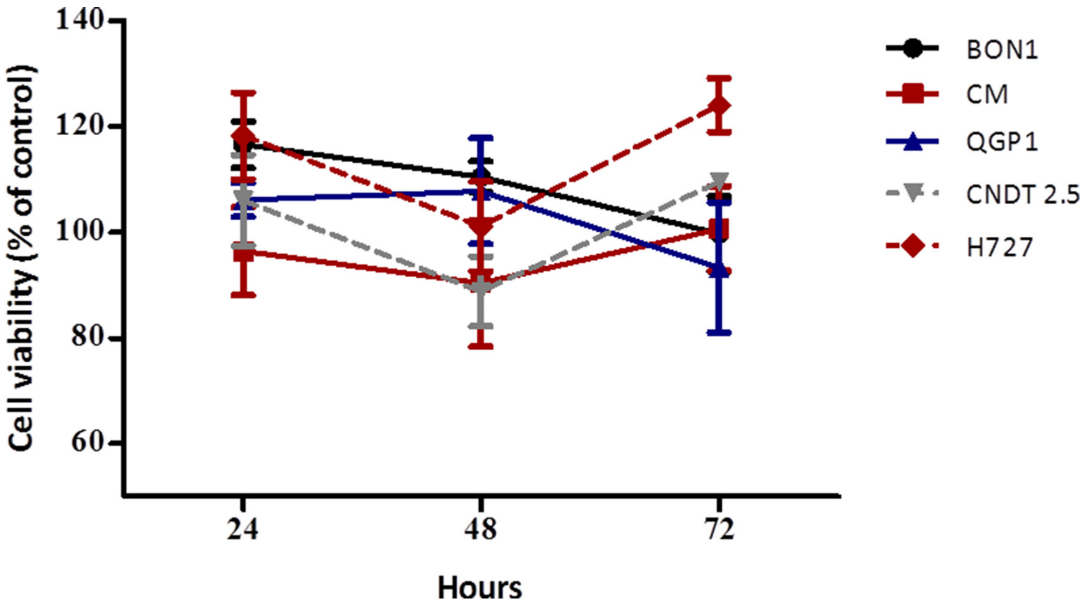

Supplementary Figure 1: Proliferation of NET cells is not influenced by CXCL12. CXCL12 at 100 ng/ml did not induce any modifications in NET.

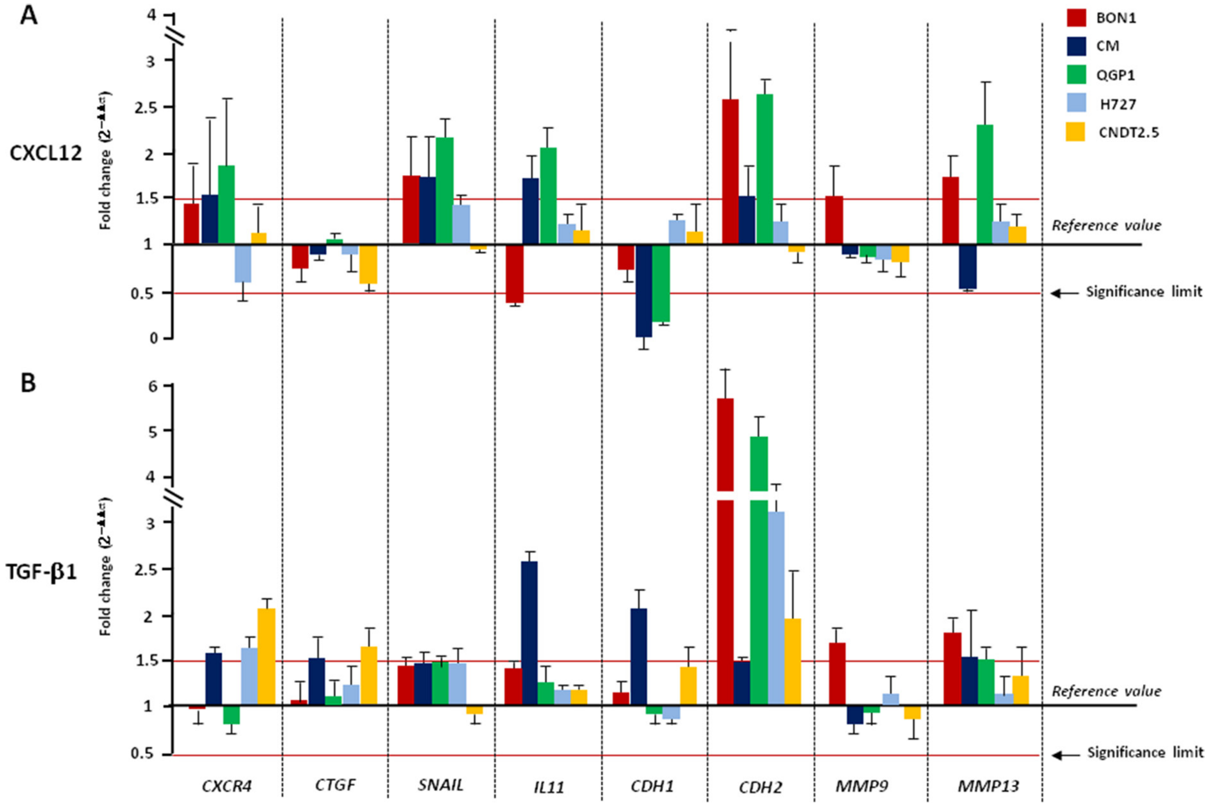

Supplementary Figure 2: CXCL12 and TGF-β1 elicit EMT. After 24 hr-incubation, both CXCL12 (**A**) and TGF-β1 (**B**) alter the EMT transcriptional profile of CXCR4^{high}/CXCL12^{low} NET cell lines, paralleling the data obtained from 2 hr-treatment experiments. Data are presented as mean ± SD, with β-actin as housekeeping gene. Statistical significance is marked by red lines.

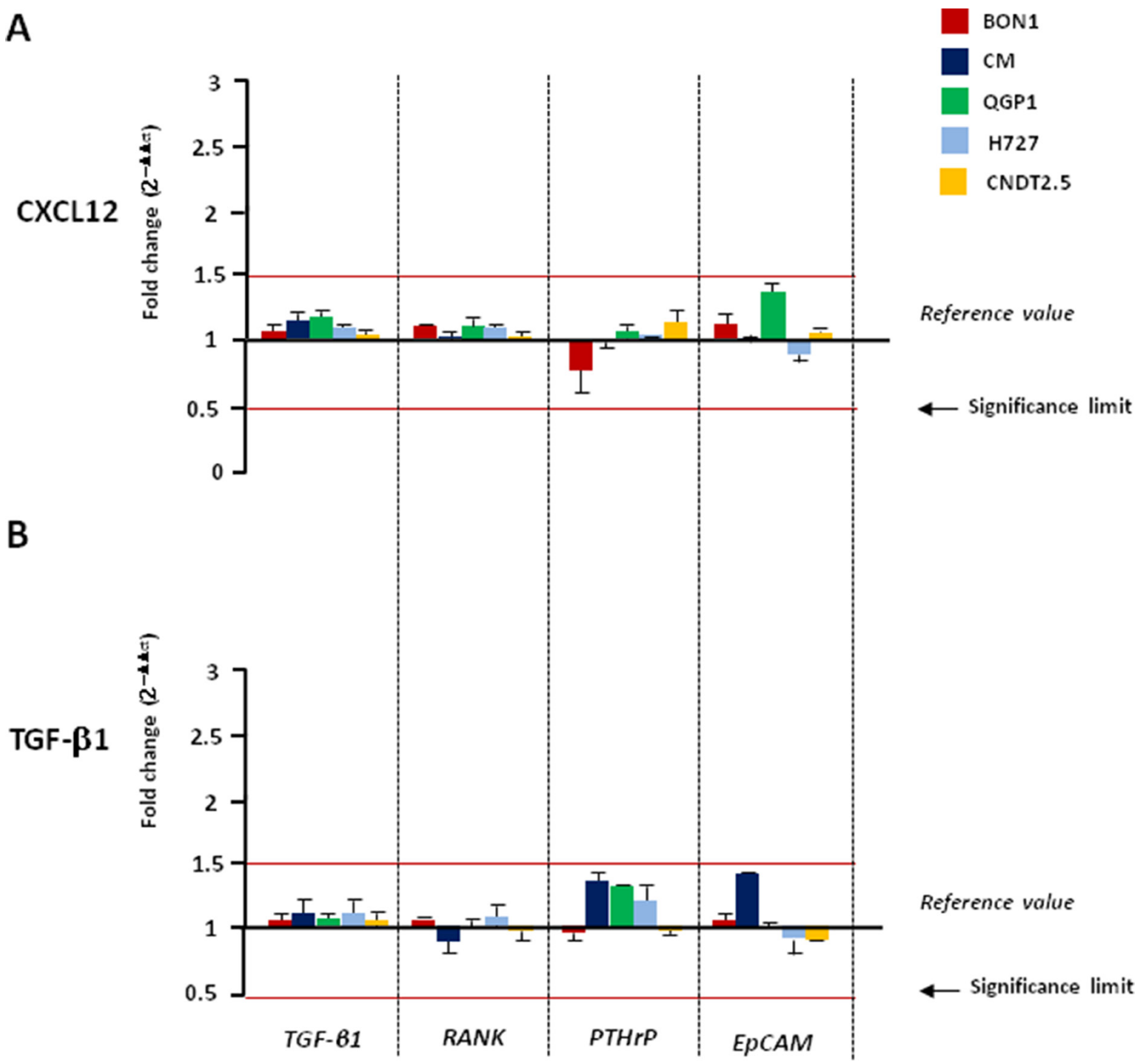

Supplementary Figure 3: CXCL12 or TGF-β1 do not affect the transcription of *TGF-β1, RANK, PTHrP*, and *EpCAM*. Incubation for 2 hours with CXCL12 (A) or TGF-β1 (B) did not significantly alter the transcript levels of *TGF-β1*, *RANK*, *PTHrP*, and *EpCAM.* Data are expressed by $2^{-\Delta\Delta CT}$ method and presented as mean ± SD. Statistical significance is marked by red lines.

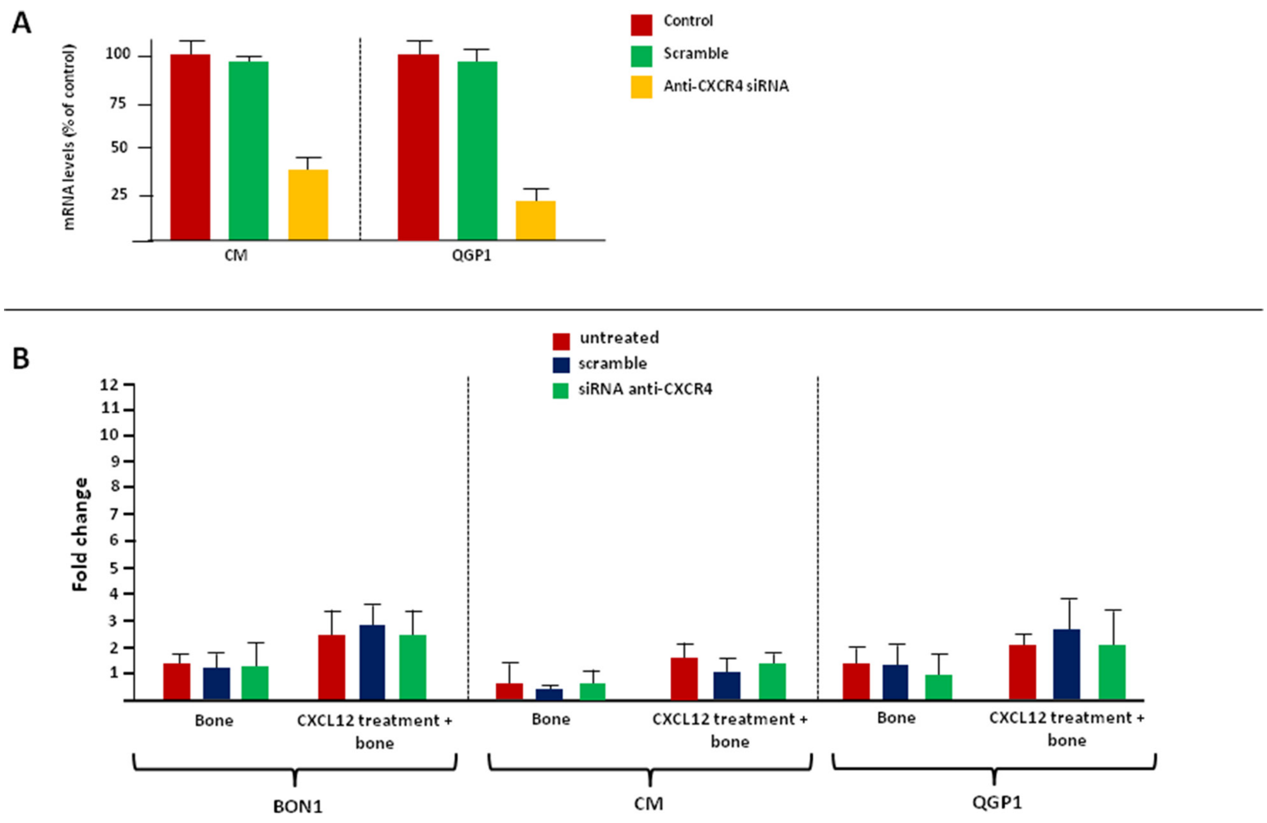

Supplementary Figure 4: Silencing of CXCR4 via siRNA is effective and downregulates the invasive properties of NET cells. (**A**) mRNA levels of CXCR4 were reduced up to 80% in cells treated with the anti-CXCR4 siRNA. (**B**) Invasiveness of the $CXCR4^{high}/CXCL12^{low}$ cell lines was suppressed after silencing of CXCR4. Data are expressed as mean ± SD, and were calculated on at least three different experiments.

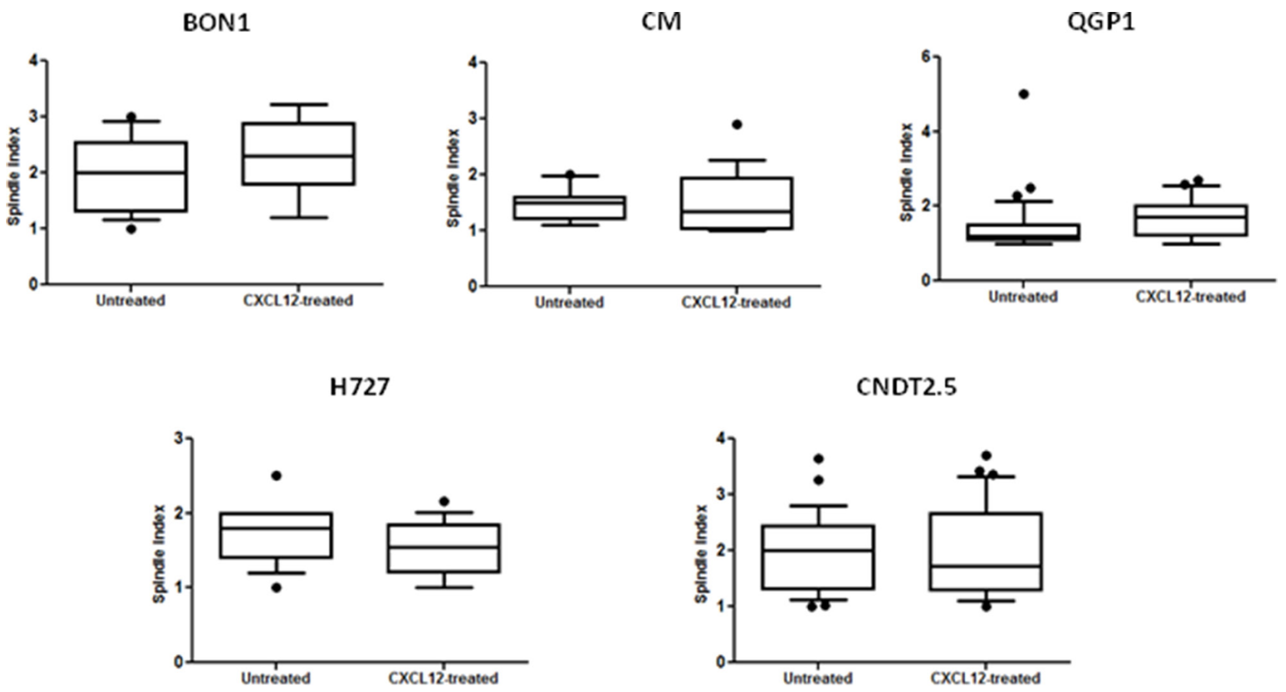

Supplementary Figure 5: Silencing of CXCR4 inhibits the morphologic modifications induced by CXCL12 in NET cells. No changes of the spindle index were observed in NET cells treated with CXCL12 following silencing of CXCR4 via siRNA. Mean, 95% confidence interval and outliers are depicted by box and whisker plots.
